# Rapid and Non-Destructive Monitoring of Moisture Content in Livestock Feed Using a Global Hyperspectral Model

**DOI:** 10.3390/ani11051299

**Published:** 2021-04-30

**Authors:** Daniel Dooyum Uyeh, Juntae Kim, Santosh Lohumi, Tusan Park, Byoung-Kwan Cho, Seungmin Woo, Won Suk Lee, Yushin Ha

**Affiliations:** 1 Department of Bio-Industrial Machinery Engineering, Kyungpook National University, Daegu 41566, Korea; uyehdooyum@gmail.com (D.D.U.); woosm7571@gmail.com (S.W.); 2 Upland-Field Machinery Research Centre, Kyungpook National University, Daegu 41566, Korea; 3 Smart Agriculture Innovation Center, Kyungpook National University, Daegu 41566, Korea; 4 Department of Biosystems Machinery Engineering, College of Agricultural and Life Science, Chungnam National University, 99 Daehak-ro, Yuseong-gu, Daejeon 34134, Korea; biosch94@gmail.com (J.K.); Santosh.sanny123@gmail.com (S.L.); 5 Department of Agricultural & Biological Engineering, University of Florida, Gainesville, FL 32611, USA; wslee@ufl.edu

**Keywords:** dairy cattle, dry matter intake, feed materials, metabolic diseases, multivariate analyses, precision feed formulation, rapid and non-destructive measurement, safe storage, total mixed ration

## Abstract

**Simple Summary:**

Moisture content is an important parameter for monitoring the quality of feed and feed materials as its established ranges serve as markers for safe storage, mixing, and feeding animals. The moisture content of feed materials changes very rapidly and necessitates rapid measurement. Current moisture content measurement methods are time-consuming, destructive, and require specialized skills. This often causes reduced and/or delayed testing, which results in the spoilage of feed and feed materials. Additionally, the improper balance of dry matter intake which is inversely proportional to moisture content often causes metabolic diseases for animals consuming the diet. To solve these, we have developed a rapid and non-destructive global hyperspectral model that could quantify moisture content in feed materials. Our results show that the developed model is robust, could provide a method to measure the distribution of moisture in feed, and has potential for implementation in a commercial setting.

**Abstract:**

The dry matter (DM) content of feed is vital in cattle nutrition and is inversely correlated with moisture content. The established ranges of moisture content serve as a marker for factors such as safe storage limit and DM intake. Rapid changes in moisture content necessitate rapid measurements. A rapid and non-destructive global model for the measurement of moisture content in total mixed ration feed and feed materials was developed. To achieve this, we varied and measured the moisture content in the feed and feed materials using standard methods and captured their images using a hyperspectral imaging (HSI) system in the spectral range of 1000–2500 nm. The spectral data from the samples were extracted and preprocessed using seven techniques and were used to develop a global model using partial least squares regression (PLSR) analysis. The range preprocessing technique had the best prediction accuracy (R^2^ = 0.98) and standard error of prediction (2.59%). Furthermore, the visual assessment of distribution in moisture content made possible by the generated PLSR-based moisture content mapped images could facilitate precise formulation. These applications of HSI, when used in commercial feed production, could help prevent feed spoilage and resultant health complications as well as underperformance of the animals from improper DM intake.

## 1. Introduction

Moisture content is an important and widely used indicator in the processing and testing of foods. The terms moisture content and water content have been used interchangeably to designate the amount of water in a product. Since the dry matter in food is inversely related to the moisture it contains, the moisture content is of direct economic importance to the processor and the consumer. Grain that contains excess water is subject to rapid deterioration from bacteria, mold growth, heating, insect damage, and sprouting [1]. In the livestock industry, moisture content of feed materials, and total mixed ration (TMR) for cattle is very important in preventing spoilage [2] and ensuring good health of the animal. In most countries, TMRs are mixed on the farm. However, in some countries such as the Republic of Korea, TMR feed is manufactured in factories, bagged, stored, and sold to farms [2]. This is because most of the raw materials for feed production are sourced internationally, which makes it difficult for small farmers (less than 50 cattle) [3] to import, store, and process by themselves. The amount of water in the TMR feed and feed materials can also be a factor to cause bacterial and fungal spoilage of feed when the temperature is conducive [4]. However, microbial spoilage could occur in environments with low moisture content at conducive temperatures [5]. In [6], the microbial activity of selected feed materials was monitored at nine different moisture contents. In that study, they established critical moisture contents between 16–46% to be favorable for microbial growth to occur depending on the microorganism and material.

DMI is a factor that must be estimated before an animal’s diet can be properly formulated. Routine monitoring of the dry matter content of feed ingredients is an important strategy in preparing TMR for dairy cattle [7].

Estimating the moisture content could also help mitigate issues of sorting TMR feed by dairy cattle. Sorting is part of the problems associated with feeding TMR to dairy cattle. To solve the problem of sorting that could cause subacute ruminal acidosis, the addition of water to dry TMR is conventionally considered to be a beneficial management practice. It has been demonstrated that reducing TMR dry matter concentration from 80 to 64% [8] results in a reduction in the extent of feed sorting against long particles and in favor of short particles, a tendency for increased Neutral Detergent Fiber intake, and higher fat content in the produced milk (3.41 vs. 3.31%) [8]. However, reducing the dry matter concentration from 57.6 to 47.9% [9] encouraged greater feed sorting and reduced DMI in TMR containing primarily forage sources. Rapid measurement of moisture content would help guide the grower to control the right amount of water content in the TMR that would prevent sorting. 

Currently, there are well-developed and commercialized non-destructive thermal sensors. In [10], thermal imaging in agriculture was reviewed. Various specifications of operational thermal sensors were provided. This study showed a wide range of thermal imaging methods that can be used in measuring the temperature of the feed or feed materials non-destructively and rapidly. However, in moisture content, the available measurement methods include the use of a capacitance sensor for nuts and grains moisture quantification [11] and a miniaturized non-destructive microwave sensor for measuring the moisture content measurement of chickpea [12]. Furthermore, the near-infrared technique has been widely applied for estimating water stress in crops [13], feed materials [14], and in grains [15]. Additionally, change in the weight of materials before and after drying using hot air ovens, microwaves, etc., is currently used for determining moisture content [16]. 

Additionally, with the considerable physical and chemical variations in the materials used for formulating feeds, multiple non-destructive sensors would be needed to measure each material. The above moisture content measurement methods of animal feed including the conventional drying technique in a hot air oven are time-consuming and destructive. The time-consuming factor could set back livestock feed production, as the materials are also susceptible to rapid change, usually occurring before the results from the current methods have been established. 

Spectroscopic methods such as mid-infrared spectroscopy (MIRS), near-infrared spectroscopy (NIRS), and Raman spectroscopy have the advantages of being rapid and non-destructive [17]. Near-infrared hyperspectral imaging [18] combines two-dimensional object visualization obtained by spectral imaging and has the potential of effectively describing constituent distribution in a sample with each pixel containing spectral information. This is added as a third dimension of values to the two-dimensional spatial image, generating a three-dimensional data cube containing any absorption, reflectance, or fluorescence spectrum data for each image pixel [19]. Hyperspectral imaging (HSI) integrates conventional imaging and spectroscopy to obtain both spatial and spectral information simultaneously from a sample at spatial resolutions varying from the level of single cells up to the macroscopic objects giving it a comparative advantage of enabling rapid and non-destructive automated screening on a large-scale [20]. Data acquired using HSI requires preprocessing to remove the noises that it usually contains. These include noise of length variation along the direction of light leading to non-linearities from the light scattering and random and scattered noise produced from the device. These noises could considerably influence the spectra and the prediction model. This makes preprocessing the data a critical step before chemometric modeling to separate important wavelength information from unnecessary redundant information contained in the data. Several spectral preprocessing methods have been explored, depending on the type and level of noise. These methods include minimum, maximum, and range normalization, multiplicative scatter correction (MSC), standard normal variate (SNV), and Savitzky-Golay (SG) 1st and 2nd derivatives methods [21]. HSI systems have been applied at paddock, plot, farm, and catchment scales to determine the type and quality of forage [22], with no information on the factory scale, as well as on heterogeneous samples like TMR and materials used in the formulation. 

Consequently, in this study, we (a) investigated and determined the moisture content of different TMRs, conventional and alternative materials using the standard oven method; (b) investigated the influence of different wavelength and preprocessing methods on prediction accuracy; and (c) developed a global model for moisture content determination irrespective of the feed or feed material.

## 2. Materials and Methods

### 2.1. Sample Preparation of TMR Feed and Feed Materials

**Figure 1:**
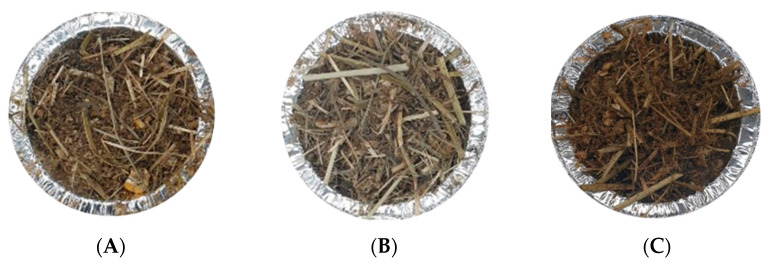
Images of the TMR feed: sample with original moisture content (**A**); sample with lower moisture content (**B**); and sample with higher moisture content (**C**).

A TMR feed sample (composed of Timothy hay: 35% DM, corn silage: 24% DM, palm-kernel expeller: 7% DM, almond pie 7% DM, rice bran: 9% DM, mushroom medium: 6% DM, soy sauce cake: 6% DM, and distillers’ dry grain: 6% DM), mixed and blended by-products (composed of palm-kernel expeller: 15% DM, almond pie: 15% DM, corn bran: 20% DM, rice bran: 15% DM, mushroom medium: 15% DM, soy sauce cake: 15% DM and distillers’ dry grain: 5% DM) and three major by-products (Palm-kernel expeller, almond pie, and corn bran) commonly used as feed materials were acquired from a factory in Gyeongju, Republic of Korea. The samples were processed specifically to alter the moisture content in a way that created three treatments for each sample as described below:

- Original TMR feed sample and feed materials (Figure 1A)
- Samples with lower moisture content than the original TMR feed and feed materials (Figure 1B)
- Samples with higher moisture content than the original TMR feed and feed materials (Figure 1C).

To achieve this, a group of samples were kept in the original condition (one sample from each group) as shown in Figure 1A. Another group was dried at 40 °C for a total duration of 9 h 30 min (Figure 1B). One sample from each group was taken out of the drying oven every 30 min (a total of 19 samples from one group) and poured into an airtight plastic bag to achieve equilibrium moisture content. The last group was rewetted with an incremental 5 mL of water to 200 g of sample (total of 20 samples from one group), mixed thoroughly, and poured into an airtight bag to achieve equilibrium moisture (Figure 1C). These resulted in a total of 40 samples with varied moisture content for each group (TMR feed and feed materials). Equations (1) and (2) were used in computing the moisture content.

The treated samples (drying and wetting) were tempered in airtight plastic bags for 24 h to achieve equilibrium moisture content.

a. The initial moisture content of the samples was determined using the standard oven method (135 °C for 2 h) referring to Equation (1).

(1) $$M_{wb}=\frac{W_{m}}{W_{m}+W_{d}}\;\;$$

where $M_{wb}$ is moisture content (MC) on wet basis (%), *W_m_* is the weight of water in the feed and feed materials and *W_d_* is the weight of dry matter in the feed and feed materials.

b. The dry matter (*DM*) in the feed was computed using Equation (2).

(2) $$DM=W_{o}\times \left(1-M_{wb}\right)$$

where $W_{o}$ is the initial weight of the feed and feed materials, and $M_{wb}$ is the initial moisture content of the feed and feed materials in decimal calculated using Equation (1). 

The difference in weight of the wetted and dried feed and feed materials before addition of water and drying and the *DM* (Equation (2)) were used in computing the new moisture content. The *W_m_* and *W_d_* were estimated by measuring the weight of the feed or feed materials before and after drying and wetting.

### 2.2. HSI Image Acquisition of TMR Feed and Feed Materials and Correction 

The samples were prepared in a circular petri dish (ϕ 90 × 15 mm) with weight depending on their densities (50–100 g). The surface of the samples was evenly distributed in the petri dish before HSI acquisition. 

**Figure 2:**
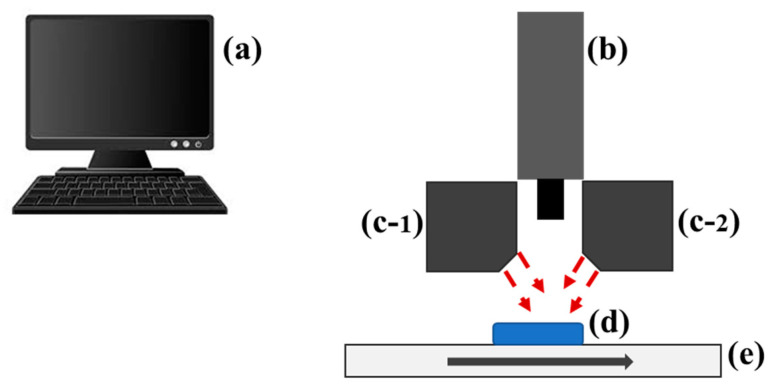
HSI system for spectral image acquisition from TMR feed samples: Computer unit (**a**); Sensing unit consisting of a lens, spectrograph, and camera (**b**); Light sources (**c**); Sample holder (**d**); and conveyor system (**e**).

A line-scan type short wave infrared hyperspectral imaging system was used in acquisition of the images of the samples as shown in Figure 2. The hyperspectral imaging system was made up of the following: (a) line scan type hyperspectral camera (Hyperspec SWIR, Headwall Photonics, Fitchburg, MA, USA), (b) C-mount lens with 25 mm f/1.4 and (c) a moving stage. The camera was operated in a spectral wavelength range of 894–2504 nm with a spectral interval of approximately 5.85 nm hence a total of 275 spectral bands. A total of six 100 W tungsten-halogen lamps (Light Bank, Ushio Inc. Tokyo, Japan) with fiber optics (three on each side) were used to illuminate feed samples. A computer programmed motorized sample stage was integrated to move the samples towards the camera Field of View (FOV). The sensing unit was linked to a computer through a frame grabber with a standard camera link cable.

The petri dishes containing feed samples were placed onto the sample holder mounted on the translation stage. The HSI data were collected with a 47 ms exposure time. The distance of the sample from the camera lens was approximately 34 cm, and the samples were measured with a scanning speed of 5.3 mm/s, and a total scan of 600 scans/sample. As the stage moved, the samples were scanned line by line in the wavelength range of 894–2504 nm. The acquired hyperspectral images were saved in a three-dimensional format containing two spatial dimensions (x and y) and a spectral dimension (*λ*). A total of 28 s was required to measure a single feed sample.

### 2.3. Calibration of Spectral Images

Calibration steps were applied to remove the dark current noise and non-uniform illumination effect from the sample images using the white and dark reference images acquired during measurement. The dark image (0% reflectance) was obtained by turning off the light source and covering the lens with a black lid, and the white image (~99% reflectance) was obtained with a white Teflon board. Thus, the normalized reflectance value was calculated using Equation (3).

(3) $$X_{C}=\frac{T_{ij}^{R}-T_{ij}^{D}\;}{T_{ij}^{W}-T_{ij}^{D}\;}$$

where $T_{ij}^{R}\;\left(\lambda \right)$ is the raw reflectance image of the feed sample, $T_{ij}^{D}\;\left(\lambda \right)$ is the dark image, $T_{ij}^{W}\;\left(\lambda \right)$ is the white image, and *X_C_* is the calibrated image. Where *i* and *j* are the pixel number and waveband, respectively.

### 2.4. Preprocessing of Spectral Images

**Figure 3:**
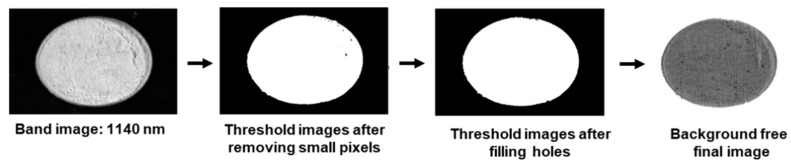
Preprocessing steps of hyperspectral image for discarding background area.

Calibrated feed sample images were preprocessed to remove background noises. This was done to acquire an image containing only the sample (Figure 3) and avoid any interference from the background [23]. For preprocessing of the image, a single waveband image was selected from the hypercube. The hyperspectral band image of 1140 nm was used to remove the irrelevant background pixels because of the highest peak of sample at this band. This was because the plotted spectra showed the highest peak near 1140 nm waveband, thus the highest difference from the background. A threshold value (0.46) was applied to turn all the sample pixels as 1 and the background pixels were turn to 0 (Figure 3). The threshold value was selected at the average value between the maximum and minimum values of the feed and feed material samples and the background pixel intensity. For perfect background removal, the final masked image was created using the morphological method (image erodes and filling) in the masked image with the primary background removed. The background free band image was then multiplied to each image in hypercube to generate a background free hypercube.

### 2.5. Pre-Processing of HSI Data Acquired from TMR Feed and Feed Materials 

Optimal data collection is the most important step in developing hyperspectral imaging models for the prediction of components. The acquired hyperspectral image spectrum contains numerous noises, such as random noise, the noise of length variation along the direction of light leading to non-linearities from the light scattering which could significantly influence the spectra, and scattered noise produced from the device. Preprocessing of spectral data is the most critical step before chemometric modeling using tools such as Partial Least Squares (PLS) and Principal Component Analysis (PCA). Consequently, it is necessary to preprocess spectra to separate important wavelength information from unnecessary redundant information contained in the data [24]. In this study, several spectral preprocessing methods were explored to correct HSI data. These include normalization methods (minimum, maximum, and range normalization), Standard normal variate (SNV), multiplicative scatter correction (MSC), and Savitzky-Golay (SG) 1st and 2nd derivatives methods.

*i*. *Multiplicative Scatter Correction (MSC)*

Multiplicative Scatter Correction (MSC) is a concept where undesirable scatter effect is removed from the data matrix preceding model development. Two steps are used in MSC (Equations (4) and (5)):

*a*. *Correction coefficient estimation*

(4) $$X_{org}=b_{o}+b_{ref,1}\;.\;X_{ref}+e$$

*b*. *Logged spectrum correction*

(5) $$X_{corr}=\frac{X_{org}-b_{o}}{b_{ref}{,}_{1}}=X_{ref}+\frac{e}{b_{ref}{,}_{1}}$$

where *X_org_* is original sample spectra, *X_ref_* is reference spectrum, *e* is the unmodeled part of *X_org_, X_corr_* is the corrected spectra, and *b*_0_ and *b_ref*,1*_* are scalar parameters.

*ii*. *Standard Normal Variate (SNV)*

Standard Normal Variate (SNV) is another method applied to scatter correction. The basic format for SNV correction is the same as that for the conventional MSC (Equation (6)).

(6) $$X_{corr}=\frac{X_{org}-a_{0}}{a_{1}}$$

where *a*_0_ is the average value of the sample spectrum to be corrected, *a*_1_ is the standard deviation of the sample spectrum.

*iii*. *Savitzky-Golay derivations*

Savtizky and Golay (SG) popularized a method for numerical derivation of a vector that includes a smoothing step. To find the derivative at center point *i*, a polynomial is fitted in a symmetric window on the raw data. When the parameters for this polynomial are calculated, the derivative of any order of this function can easily be found analytically, and this value is subsequently used as the derivative estimate for this center point. This operation is applied to all points in the spectra sequentially. The number of points used to calculate the polynomial (window size) and the degree of the fitted polynomial are both decisions that need to be made. The highest derivative that can be determined depends on the degree of the polynomial used during the fitting (i.e., a third-order polynomial can be used to estimate up to the third-order derivative).

### 2.6. Development of Moisture Content Prediction Models for TMR Feed and Feed Materials 

The preprocessed spectra were used to develop the partial least squares regression (PLSR) model. PLSR is a multivariate analysis method used to assess the correlation between various independent variables (X) and dependent variables (Y) [25]. Since a bad signal-to-noise ratio (SNR) was observed for the wavelengths above 1917 nm, because of the sensitivity of the detector, we selected the waveband range between 894–1917 nm (175 bands) excluding the noisier spectral region. The prediction after this wavelength (1917 nm) is poor and not robust. Subsequently, the wavelengths that have a major influence on moisture content prediction were selected based on the beta coefficient obtained from developed PLS model. Additionally, the significant band for moisture content prediction in this study was below the 1917 nm bands as also reported in [26,27]. The efficiency of PLS regression model was evaluated based on the prediction accuracy (R^2^) and standard errors for calibration, cross-validation, and prediction. Inappropriate numbers of latent variables selection can cause under- or over-fitting, leading to suppression of spectral information, incorrect model interpretation, and spectral noise in the regression model. Consequently, the optimal number of latent variables was selected based on the lowest value of predicted root mean square error (RMSE) by the leave-one-out cross-validation process during the cross-validation (CV) process.

PLS regression was implemented as multivariate analysis and regression method to determine the linear models of prediction between the spectral data (X-matrix, N_samples_ × K_wavelengths_) and the values of the parameters obtained from the reference measurement (Y-matrix, N_samples_ × 1). The linear relationship between X and Y is predicted using Equations (7) and (8).

X = TP^T^ + E (7)

Y = UQ^T^ + F (8)

where Y is the matrix of dependent variables conforming to the sample values measured from the reference data obtained using standard oven methods and calculations in Equations (1) and (2). X is the *n* × p matrix of independent variables corresponding to the spectral variables for each hyperspectral measurement. The matrix X decomposes into the loading matrix P, score matrix T, and error matrix E. The matrix Y decomposes into the loading matrix Q, score matrix U, and error matrix F. 

Furthermore, the entire X and Y matrix data were divided into calibration and validation sets, which consisted of 70% of the data for calibration and 30% for validation. 

### 2.7. Model Evaluation of TMR Feed and Feed Materials

The developed models were evaluated using several statistical parameters. This includes coefficient of determination shown in Equation (9). The coefficient of determination encompasses calibration (R^2^C), prediction (R^2^P), and cross-validation (R^2^CV). Other statistical parameters were the standard error of calibration (SEC), prediction (SEP), and cross-validation (SEV).

(9) $$R^{2}=\frac{\sum_{i=1}^{n}\;{\left(y_{i}-{Ŷ}_{i}\right)}^{2}}{\sum_{i=1}^{n}\;{\left(y_{i}-Ȳ\right)}^{2}}$$

where the predicted and measured components in TMR feed and feed materials are Ŷ*_i_* and *y_i_*, respectively. The number of validation sets observations is denoted with *n* and the mean of measured values is denoted with Ȳ.

### 2.8. Image Visualization and Moisture Content Distribution Map of Samples

The moisture content of the TMR feed and feed materials were computed for each pixel to visualize the corresponding distribution made possible with each pixel in a hyperspectral image possessing a spectrum. Although it is practically impossible to obtain the precise quality parameters of every pixel within a sample by chemical analysis, it could be predicted by the optimal calibration model. The hyperspectral image was unfolded into a two-dimensional (2D) matrix and then multiplied by the regression (beta) coefficient obtained from the best calibration model and applied to the selected wavelengths. The resultant vector was then folded back to form a 2D image. A median filter of 3 × 3 was applied to the 2D image for enhancing image quality for visual display. The difference in the predicted attributes within one sample and those from other sources can be visualized from the generated 2D images. 

**Figure 4:**
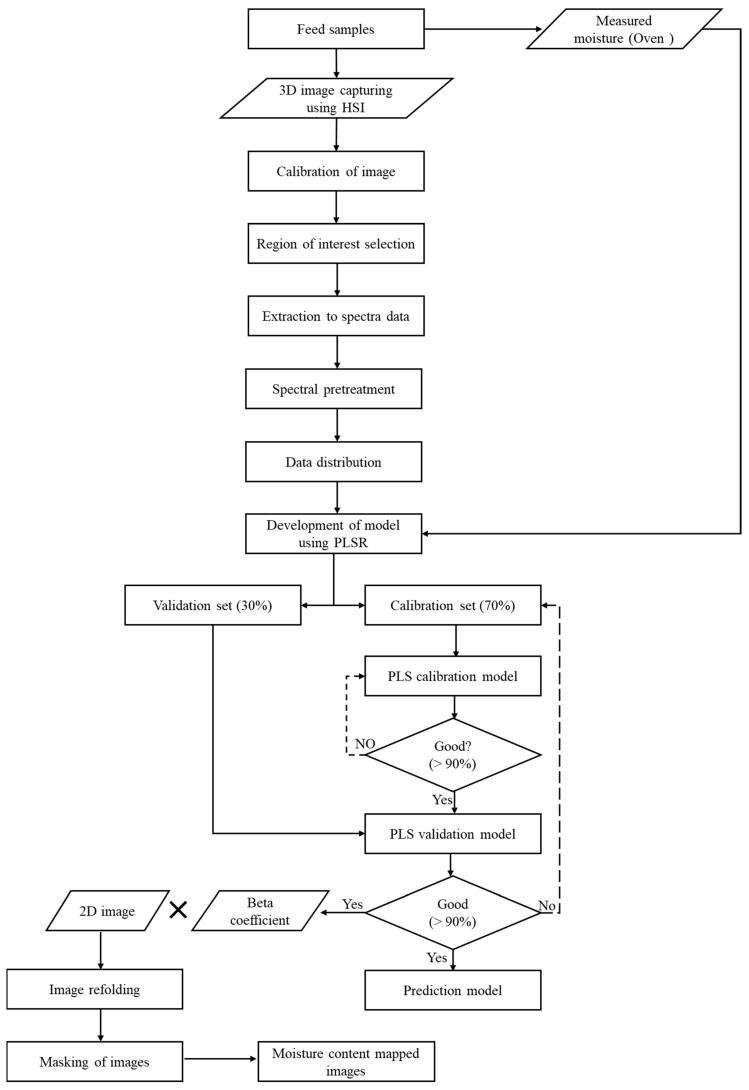
Steps used in processing hyperspectral images and developing moisture content prediction models regardless of the type of feed and feed materials.

The steps of data preprocessing, prediction model development, and generating concentration maps are shown in Figure 4. The algorithms were implemented in MATLAB, version 2020a (MathWorks, Natick, MA, USA). 

## 3. Results and Discussion 

The computed moisture content with initially measured values using the oven drying method ranged from 13 to 78% for the TMR feed (S1), 5 to 54% for the mixed by-products (S2), 1.59 to 76.41% for palm kernel expeller (S3), 2.25 to 62.42% for almond pie (S4) and 4.43 to 52.21% for corn bran (S5) (Table A1 in Appendix A).

### 3.1. HSI Features of TMR Feed and Feed Materials 

**Figure 5:**
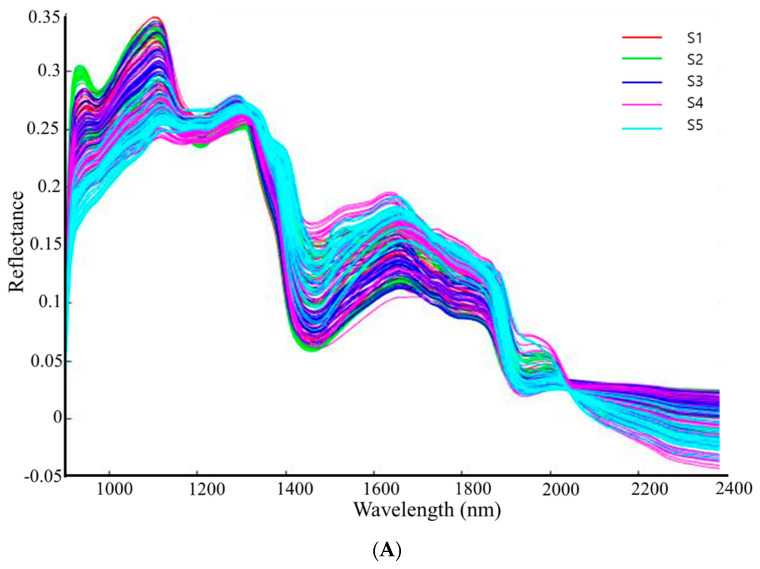

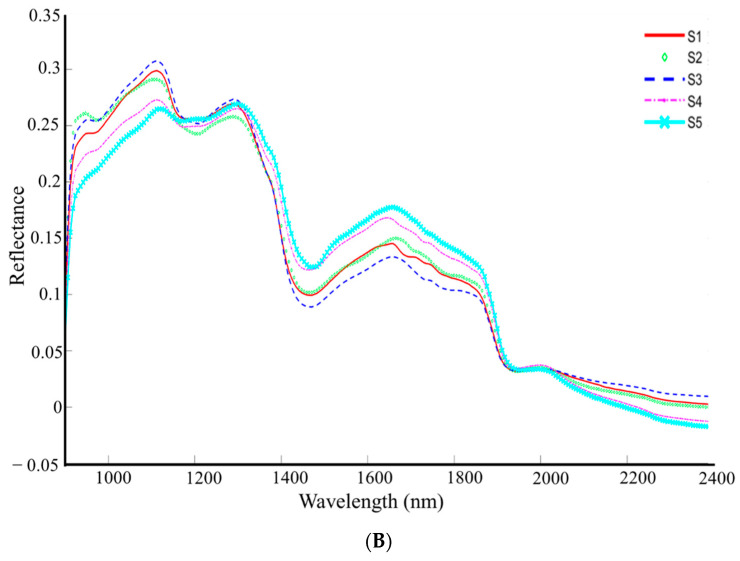
MSC preprocessed spectra (**A**) and mean spectra (**B**) of TMR feed and by-product samples (n = 200) for moisture content prediction (S1: TMR; S2: mixed by-products; S3: palm kernel expeller; S4: almond pie; S5: corn bran).

The MSC preprocessed spectra (Figure 5A) and mean spectra (Figure 5B) for all the samples (n = 40 samples × 5 samples) are shown in Figure 5. The spectral region (894 nm–1917 nm) used is related to various peaks in broadband such as the O-H, C-H, and N-H functional groups. The disparities in spectral pattern appeared particularly around 1150 nm–1250 nm, and 1400 nm–1750 nm. In addition, the peaks around 1225 nm and 1420 nm are associated with the C-H and O-H overtones, respectively. The small peak at 1530 nm is associated with the N-H stretch first overtone and 1660 nm to the aromatic C-H stretch first overtone [28]. These represent the variation in the moisture, carbohydrate, and protein contents of the samples [28,29].

### 3.2. Prediction of Moisture Content in the Samples

**Figure 6:**
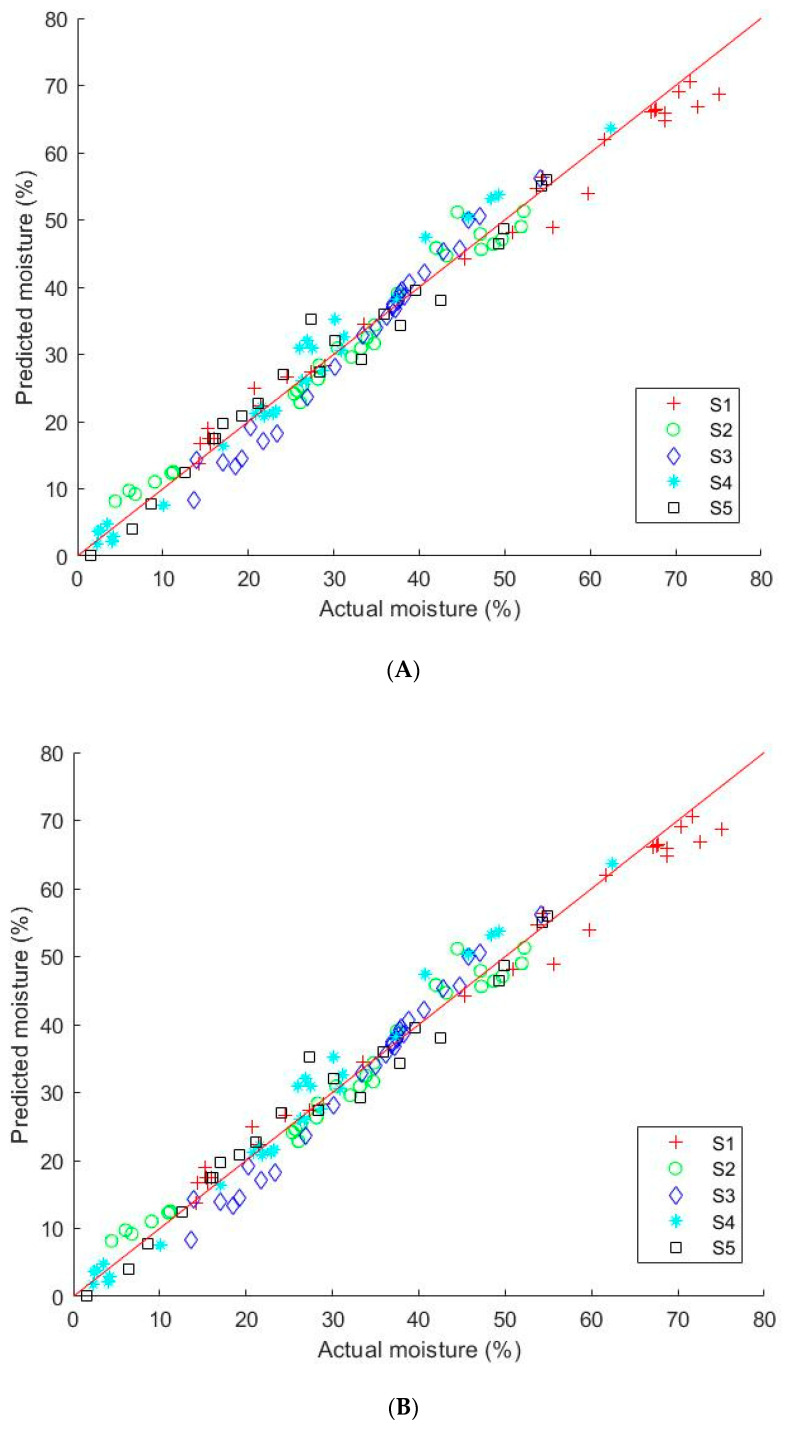
Moisture content prediction plot: (**A**) Calibration plot; (**B**) Prediction plot (S1: TMR; S2: mixed by-products; S3: palm kernel expeller; S4: almond pie; S5: corn bran).

Table 1 shows the results obtained from PLSR model developed with each preprocessing method. All methods showing a good prediction accuracy (R^2^) of over 93% and prediction errors (SEP) of less than 5%. The PLSR prediction values for moisture content in multiple by-product samples are shown in Figure 6, where most of the predicted values fall near the line of best fit.

Preprocessing methods and partial least squares regression multivariate technique and were applied to the acquired spectra. We used the spectral data in the wavelength range between 894 nm and 1917 nm to build the models as the HSI system used in this study produces noisy spectra above 1917 nm. From the data preprocessing methods in Table 1, the range preprocessing method generated the maximum correlation coefficient values with R^2^C of 0.98, R^2^V of 0.97, and R^2^P of 0.98. It also had the least SEP of 2.59%. However, all other methods were also satisfactory, with less than 5% SEP values. The optimal number of latent variables was determined from minimum SEV values in the cross-validation process. 

**Figure 7:**
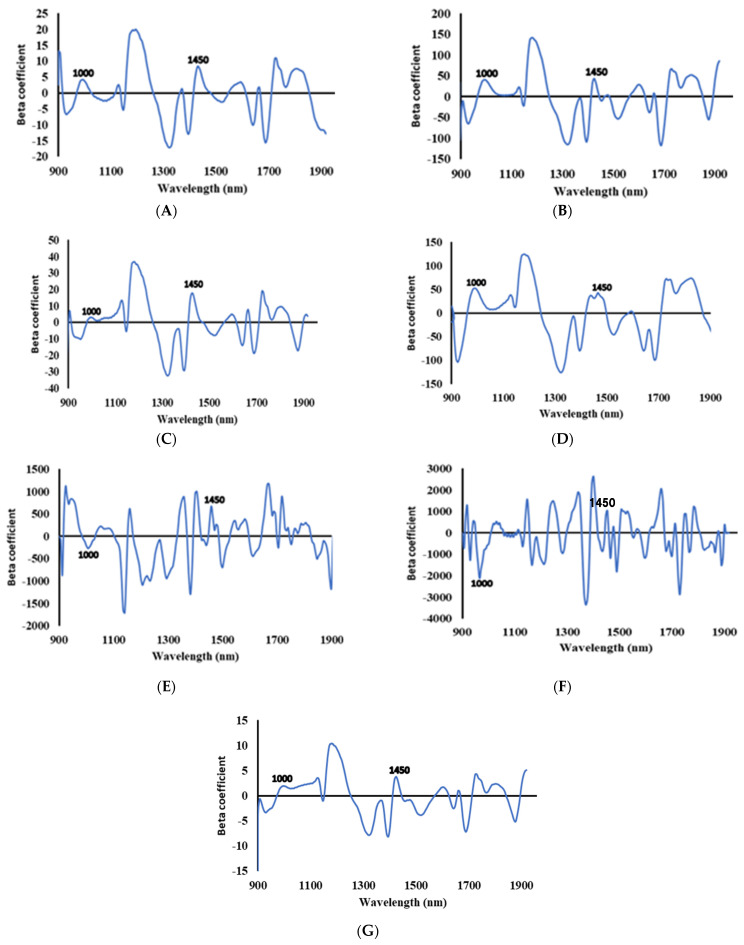
Beta coefficient plot: mean preprocessing (**A**); MSC preprocessing (**B**); range preprocessing (**C**); raw (**D**); Savitzky Golay 1st preprocessing (**E**); Savitzky Golay 2nd preprocessing (**F**); and SNV preprocessing (**G**).

Overtones and combinations of fundamental vibrations of molecules comprising -OH, -NH, and -CH groups are characterized in the near-infrared (NIR) spectrum. These would absorb based on the component such as moisture content, protein, etc. [30]. The broadband peaks in the spectrum are shown in the beta coefficient graph for all the preprocessing methods in Figure 7. In Figure 7E,F, the Savitzky Golay 1st and 2nd preprocessing methods showed that the highest positive peaks were obtained around 1660 nm and 1400 nm, respectively. However, these were not the important peaks in the developed model. The considerable amount of other nutrients contained in the TMR feed and feed materials such as carbohydrate and protein resulted in high absorption peaks. These components also increased with the reduction in moisture content as the measured components were in percentages.

The important absorption peaks appeared around 1000 nm and 1450 nm in most of the preprocessing methods, with the exception of SNV preprocessing, where the peak at 1000 nm was not very conspicuous. The peak around 1000 nm is related to the O-H stretching second overtone, representing the moisture content in the samples [31]. The peak at 1450 is associated with the first overtone of O-H stretching indicating the moisture content absorption in the samples [32]. These indicate that all the models are robust and can accurately predict moisture content in the TMR feed and feed materials. 

### 3.3. Imaging of Moisture Content in TMR Feed and Feed Materials

**Figure 8:**
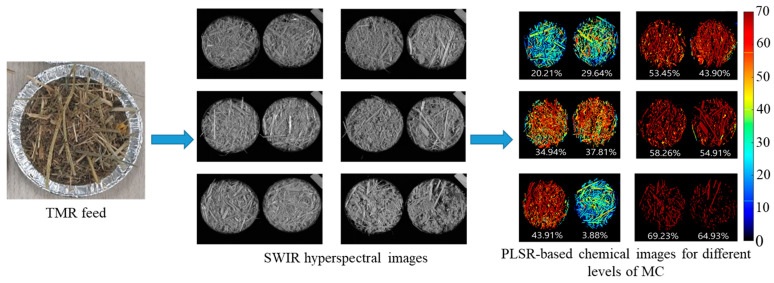
Original TMR feed, SWIR hyperspectral images of 1140 nm, and PLSR-based images for different levels of moisture content in TMR feed.

Every pixel in the hyperspectral image has its unique spectrum. As a result, the moisture content can be computed with the spectrum of any pixel in the sample. To compute the moisture content of the whole sample, all spatial pixels of the hyperspectral image should be considered. In Figure 8, the original TMR feed, SWIR hyperspectral images, and PLSR-based images for different levels of moisture content are shown. The images were developed by multiplying the obtained beta coefficient (regression coefficient) from the PLSR model with the spectra of each pixel in the image. In the generated moisture content images, the moisture content in the TMR feed was unevenly spread. The disparate colors (Figure 8) correspond to levels of moisture in the TMR feed and are proportional to the spectral differences of the individual pixels. This is because of factors such as exposure of a portion of the feed to the open environment that allows evaporation or absorption of moisture depending on the temperature and relative humidity of the storage environment and other substrates in the feed. This stops the feed from reaching an equilibrium state. These moisture maps offer rapid and easy access to the spatial distributions in which the relative intensities are indicated by the color bar. These acquired distribution maps validate the benefits of HSI in analyzing heterogeneous samples like TMR feed. This result cannot be achieved with conventional imaging or spectroscopy techniques.

Consequently, this study showed the potential of using HSI to estimate the moisture content of TMR feed and feed materials. 

### 3.4. Models for Feed Moisture Composition Measurement

Two models were proposed for manufacturing TMR feed with accurate moisture content. Moisture content is a very important parameter in the feed. It plays a vital role in the storability and, most significantly, the proliferation of dangerous microorganisms such as Aspergillus species that are responsible for the production of toxins such as Aflatoxin B1, as discussed in the background. Furthermore, the moisture content is inversely proportional to dry matter, which is an important component in formulating the diet of dairy cattle. The current methods to estimate the moisture content of feed and feed materials are destructive and time-consuming. In this research, we demonstrated the possibilities of using hyperspectral imaging technology for the rapid and non-destructive scans of all feed samples passing through the conveyor belt for safe production, storage, and feeding of livestock. 

**Figure 9:**
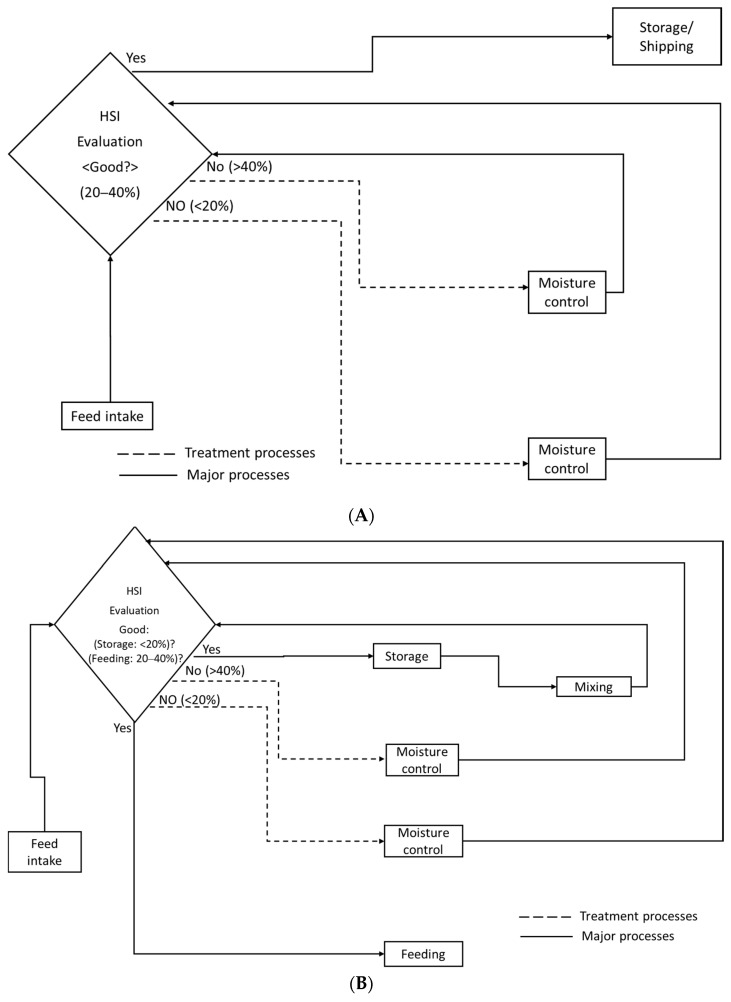
Model for production of TMR feed with precise moisture content and DMI in the factory (**A**); on the farm (**B**).

In the first proposed model (Figure 9A), since the hyperspectral imaging system is a line-scan and good for a conveyor belt system, it is installed to scan the TMR feed as it is conveyed before packaging, then storage and transportation to the farm. If the feed has an unsatisfactory moisture content, it will be diverted for retreatment using any method for water control depending on available resources and location in case of higher or lower moisture contents. The moisture content will be dependent on the class of animals being fed. After the retreatment process, the feed is re-evaluated each time until an acceptable standard is met. 

In the second proposed model (Figure 9B), which is on the farm, the hyperspectral imaging system is installed to examine the feed on arrival on the farm. After line-by-line examination and dependent on the class of the animal [33] and the type of feed material or feed, a decision is made which includes for example moisture control when:

- Moisture content greater than 40%;
- Moisture content below 20%, which is specific to the feed material for storage.

In the farm situation, moisture content below 20% will be acceptable for storage since feed is most likely to be stored longer on the farm. Furthermore, mixing with by-products or roughages would be carried out after storage. The mixing can be used to balance the moisture content at this stage. However, after the mixing, if the moisture content falls below the acceptable feed standard, the TMR feed moisture must be controlled in case of more than 40% or less than 20%. This process is repeated until the acceptable standard is achieved. When the moisture content is between 20 and 40%, it is within the acceptable feeding range for the stage of growth of the dairy cattle considered in this study which implies no treatment is required. This is because, in the Republic of Korea, different stages of growth of dairy cattle necessitate the moisture of the feed being controlled to a set percentage. 

In both models (Figure 9A,B), one hyperspectral imaging system is proposed to produce safe and satisfactory feed. Furthermore, the speed of the conveyor belt in TMR factories is within the scanning speed of the hyperspectral imaging system, suggesting it could be implemented in a commercial setting. However, high computing power would be needed to process the data in real-time.

## 4. Conclusions

A rapid and non-destructive global model that could measure the moisture content and invariably dry matter content irrespective of feed material type using a hyperspectral imaging system was proposed and developed. The developed partial least squares regression (PLSR) models using different preprocessing techniques yielded acceptable prediction accuracies (R^2^p) of above 0.93 and standard error of prediction (SEP) of less than 5%. However, the range preprocessing technique had the best R^2^p (0.98) and SEP (2.59%). Additionally, the visual assessment in the distribution of moisture content made possible by the generated PLSR-based moisture content mapped images could facilitate precise feed formulation. The proposed approach eliminates the extensive preparation of samples, time-consuming repetitive scans, and expertise required for conventional oven and spectroscopy procedures. Our results demonstrate that the developed model is robust, and that it could provide a method to assess the distribution of moisture in feed, while having the potential for implementation in a commercial setting. This would help prevent feed and feed material spoilage and resultant health complications, the underperformance of animals from the improper intake of dry matter, and the associated financial losses.

## Figures and Tables

**Table 1:** Results obtained with global PLSR model for prediction of moisture content in multiple by-products.

Method	*n* of Calibration Set	*n* of Validation Set	LV ^a^	Calibration ^b^		Cross-Validation ^c^		Prediction ^d^	
				R^2^C ^e^	SEC ^f^ (%)	R^2^V ^g^	SEV ^h^ (%)	R^2^P ^i^	SEP ^j^ (%)
Mean ^k^	140	60	10	0.98	2.61	0.97	2.83	0.98	2.76
Max ^l^	140	60	10	0.98	2.49	0.97	2.82	0.98	2.63
Range ^m^	140	60	10	0.98	2.62	0.97	2.92	0.98	2.59
MSC ^n^	140	60	10	0.97	2.90	0.97	3.18	0.97	2.86
SNV ^o^	140	60	10	0.98	2.76	0.97	3.04	0.98	2.68
Savitzky Golay (1st) ^p^	140	60	10	0.97	2.99	0.96	3.47	0.97	3.31
Savitzky Golay (2nd) ^q^	140	60	10	0.94	4.19	0.93	4.81	0.93	4.58
Raw ^r^	140	60	10	0.96	3.68	0.95	4.07	0.95	4.11

^a^ LV: Latent variable; ^b^ Calibration (comparison of a known standard measured values and measurement using I); ^c^ Cross-validation (model evaluation with independent data set to test performance); ^d^ Prediction (estimation of the quantity of moisture using the developed models); ^e^ R^2^C: Coefficient of determination for calibration; ^f^ SEC: standard error of calibration; ^g^ R^2^V: coefficients of determination for cross-validation; ^h^ SEV: standard error of cross-validationI; ^i^ R^2^P: Coefficient of determination for prediction; ^j^ SEP: standard error of prediction; Preprocessing methods (^k^ Mean; ^l^ Max; ^m^ Range; ^n^ Multiplicative Scatter Correction (MSC); ^o^ Standard Normal Variate (SNV) and ^p^ Savitzky-Golay 1st derivation; ^q^ Savitzky-Golay 2nd derivation) and ^r^ Raw data model (Raw).

## Data Availability

Not applicable.

## Appendix A

**Table A1:** Moisture content of TMR feed and feed materials.

Sample Number	TMR (%)	Mixed By-Products (%)	Palm-Kernel Expeller (%)	Almond Pie (%)	Corn Bran (%)
1	50.84	37.44	15.84	13.87	29.60
2	46.26	36.55	39.57	17.02	27.30
3	68.77	41.95	49.90	19.20	28.16
4	67.52	44.70	20.20	20.33	26.32
5	64.37	40.61	23.71	21.59	25.71
6	67.05	37.98	49.23	22.91	25.38
7	67.07	38.23	16.13	23.27	26.07
8	75.12	37.16	42.52	21.85	47.84
9	62.10	35.05	25.58	26.29	47.21
10	55.69	33.37	27.34	26.35	48.70
11	54.39	37.79	28.34	27.52	30.42
12	61.69	36.12	33.20	26.69	28.27
13	68.80	40.32	37.73	30.30	34.76
14	67.65	39.76	21.14	31.15	33.88
15	72.57	37.52	12.63	26.32	33.17
16	71.73	36.91	20.62	30.93	34.35
17	59.74	42.83	19.26	16.98	36.39
18	58.29	48.64	53.22	18.73	32.08
19	78.69	47.05	19.67	25.95	47.24
20	70.42	32.93	9.92	11.28	14.14
21	13.18	34.59	30.29	10.13	52.21
22	15.43	37.01	16.97	5.50	44.45
23	24.47	45.78	3.69	4.26	49.67
24	25.04	54.99	54.36	3.41	48.71
25	58.65	38.75	24.12	20.87	43.17
26	15.19	36.95	57.99	37.41	34.72
27	14.39	37.64	8.58	40.97	47.14
28	14.21	34.18	63.95	45.75	49.69
29	28.89	54.13	1.59	62.42	45.75
30	15.49	49.29	35.82	49.27	51.90
31	20.72	23.30	49.03	4.12	6.04
32	52.59	31.26	76.41	2.25	4.43
33	54.71	26.15	1.71	2.70	9.07
34	45.31	30.07	2.97	2.47	6.76
35	48.03	26.88	2.64	28.68	4.49
36	53.69	5.96	24.34	32.77	4.68
37	33.51	13.69	54.95	40.80	11.22
38	16.02	18.56	6.34	48.33	11.02
39	24.31	21.80	30.15	30.13	9.78
40	21.43	19.27	4.54	26.96	12.96
